# A novel look at the posterior rotator interval of the shoulder

**DOI:** 10.1002/jeo2.70688

**Published:** 2026-03-18

**Authors:** Elle Põldoja, Tiina Tikk, Jüri‐Toomas Kartus, Jon Karlsson, Imke Weyers, Ivo Kolts, Madis Rahu

**Affiliations:** ^1^ Department of Anatomy University of Tartu Tartu Estonia; ^2^ Department of Orthopedics NU Hospital Group Trollhättan Sweden; ^3^ University of Gothenburg Gothenburg Sweden; ^4^ Department of Orthopedics Sahlgrenska University Hospital, Sahlgrenska Academy, University of Gothenburg Gothenburg Sweden; ^5^ Institute of Anatomy University of Lübeck Lübeck Germany; ^6^ Department of Sports Medicine and Rehabilitation University of Tartu Tartu Estonia

**Keywords:** glenocapsular ligament, glenohumeral joint capsule, posterior rotator interval, rotator cuff tendon, shoulder joint

## Abstract

**Purpose:**

The capsular structures of the posterior rotator interval have not been fully described yet. Moreover, their clinical importance is still being questioned. The purpose of this study was to investigate and in detail report which part of the glenocapsular ligament is located within the posterior rotator interval.

**Methods:**

Seventeen fixed cadaveric shoulder specimens from 6 females and 11 males, with a mean age of 72.7 years (±7.2), were included. All specimens were finely dissected to reveal the superficial and deep structures of the posterior rotator interval.

**Results:**

The posterior rotator interval is visible as a distinct space between the supraspinatus and infraspinatus muscles medially, while the tendons and joint capsule fuse laterally. The superficial and deep structures located in the posterior rotator interval are in line with the scapula. The capsular fibres of the glenocapsular ligament are divided into two distinct parts, that is, the posterosuperior and mediosuperior parts. The mediosuperior part varies in shape, and in seven of seventeen cases it was absent, but the posterosuperior part was found to be constant in all specimens. The posterosuperior part of the glenocapsular ligament is located in the posterior rotator interval, attaching medially to the supraglenoid tubercle/scapular neck and laterally to the semicircular humeral ligament (rotator cable) between the middle and inferior facets of the greater tubercle of the humerus.

**Conclusion:**

The posterosuperior part of the glenocapsular ligament was shown to be a constant connective tissue structure of the posterosuperior capsule of the shoulder joint, located in the posterior rotator interval. The glenocapsular ligament may be of potential clinical relevance as an anatomical reference, serving as a posterior landmark for superior capsule restoration and as an anterior landmark for supraspinatus and infraspinatus tendon restoration in large rotator cuff tears.

**Level of Evidence:**

NA.

**Abbreviations**
CGL: coracoglenoidal ligament
CHL: coracohumeral ligament
CP: coracoid process
GCL: glenocapsular ligament
GT: greater tubercle
IS: incisura scapulae
ISP: infraspinatus muscle
LT: lesser tubercle
SCHL: humeral semicircular ligament
SSP: supraspinatus muscle
ST: supraglenoid tubercle
STSL: superior transverse scapular ligament
sup: superior part
Tmaj: teres major muscle
Tmin: teres minor muscle

## INTRODUCTION

The shoulder joint is a complex anatomical structure. To date, there is abundant information about the bony anatomy of the shoulder and rotator cuff tendons [3, 14]; however, less is known about the posterosuperior part of the shoulder joint capsule and the posterior rotator interval.

According to Miller et al. [12] and Huri et al. [5], there are two different rotator intervals: the anterior and posterior intervals. Both intervals are associated with the supraspinatus muscle, either with the anterior or posterior margin of the muscle.

The anterior rotator interval is a triangular anatomical region between the supraspinatus and subscapularis tendons and coracoid process medially [10, 13, 20]. This interval includes the coracohumeral and superior glenohumeral ligaments and the tendon of the long head of the biceps brachii [6, 8, 9]. It contributes significantly to glenohumeral joint and biceps tendon stability through the biceps pulley system and is an important surgical landmark when it comes to rotator cuff repair [1, 4, 11, 15, 19, 21].

The morphology of the posterior rotator interval has rarely been studied despite its potential surgical importance. According to Miller et al. [12], the posterior rotator interval is a clear and constant structure defined as a region between the supraspinatus and infraspinatus muscles. Medially, it consists of the glenohumeral joint capsule and laterally the tendons of the supraspinatus and infraspinatus muscles, where they fuse to insert into the greater tuberosity. According to Miller et al. [12], the anatomy of the posterior rotator interval is important when it comes to rotator cuff tendon repair. Releasing the posterior rotator interval may also be necessary to obtain an anatomic repair or closure of the posterior rotator interval to decrease the tension of the repair of the rotator cuff. Kolts [7] was the first to describe the glenocapsular ligament as a distinct connective tissue structure located between the semicircular humeral ligament (rotator cable) and glenoid cavity. Põldoja et al. [16] subsequently provided a detailed characterization of the different parts of this ligament and its blood supply. To date, the presence of this ligament within the posterior rotator interval has not been described in detail in the literature.

Therefore, the purpose of this anatomical study was to investigate and describe which part of the glenocapsular ligament is located within the posterior rotator interval.

## MATERIALS AND METHODS

The study was carried out using 17 alcohol–formalin–glycerol‐fixed cadaveric shoulder specimens (11 males and 6 females). The mean age at the time of death was 72.7 (±7.2) years (range: 61–81). Out of 17 specimens, 16 were paired, and 1 was unpaired. None of the specimens showed macroscopic signs of rotator cuff rupture. The anatomical shoulder specimens were collected from voluntarily donated bodies. The ethical approval was received from ‘Gesetz über das Leichen, Bestattungs‐ und Friedhofwesen des Landes Schleswig˗Holstein vom 04.02.2005, Abschnitt II, §9 (Leichenöffnung, anatomisch)’.

### Dissection

All 17 fixed shoulders were dissected using a dissection microscope.

#### Superficial structure dissection

The muscles of the shoulder girdle, the deltoid and pectoral muscles, were removed to the extent required for inspection of the rotator cuff. The acromion was excised from the spina of the scapulae and removed together with the coracoacromial ligament, and the posterior rotator interval was exposed. The rotator cuff tendons were cleaned of the remains of the subacromial bursa and separated from the joint capsule, and each tendon was dissected individually. The bony insertion areas of the supraspinatus and infraspinatus tendons were left intact.

#### Deep structure dissection

Thereafter, the capsule of the superior part of the shoulder joint was meticulously dissected to identify all ligamentous/soft tissue structures at the posterior rotator interval. The coracohumeral and coracoglenoidal ligaments were dissected in fine detail; the coracohumeral ligament was carefully separated during preparation from the coracoglenoidal ligament to the semicircular ligament. The coracoid process was cut at its base and moved together posteriorly with the ligaments. Subsequently, precise dissection of the glenocapsular ligament was performed along the course of macroscopically visible parallel running collagen fibre bundles, proceeding medially towards the lateral semicircular humeral ligament.

Photographs were taken throughout the dissection, and the ligament structures were carefully documented.

Since the authors performed a detailed descriptive anatomic study, no statistical analysis was performed.

## RESULTS

**Figure 1:**
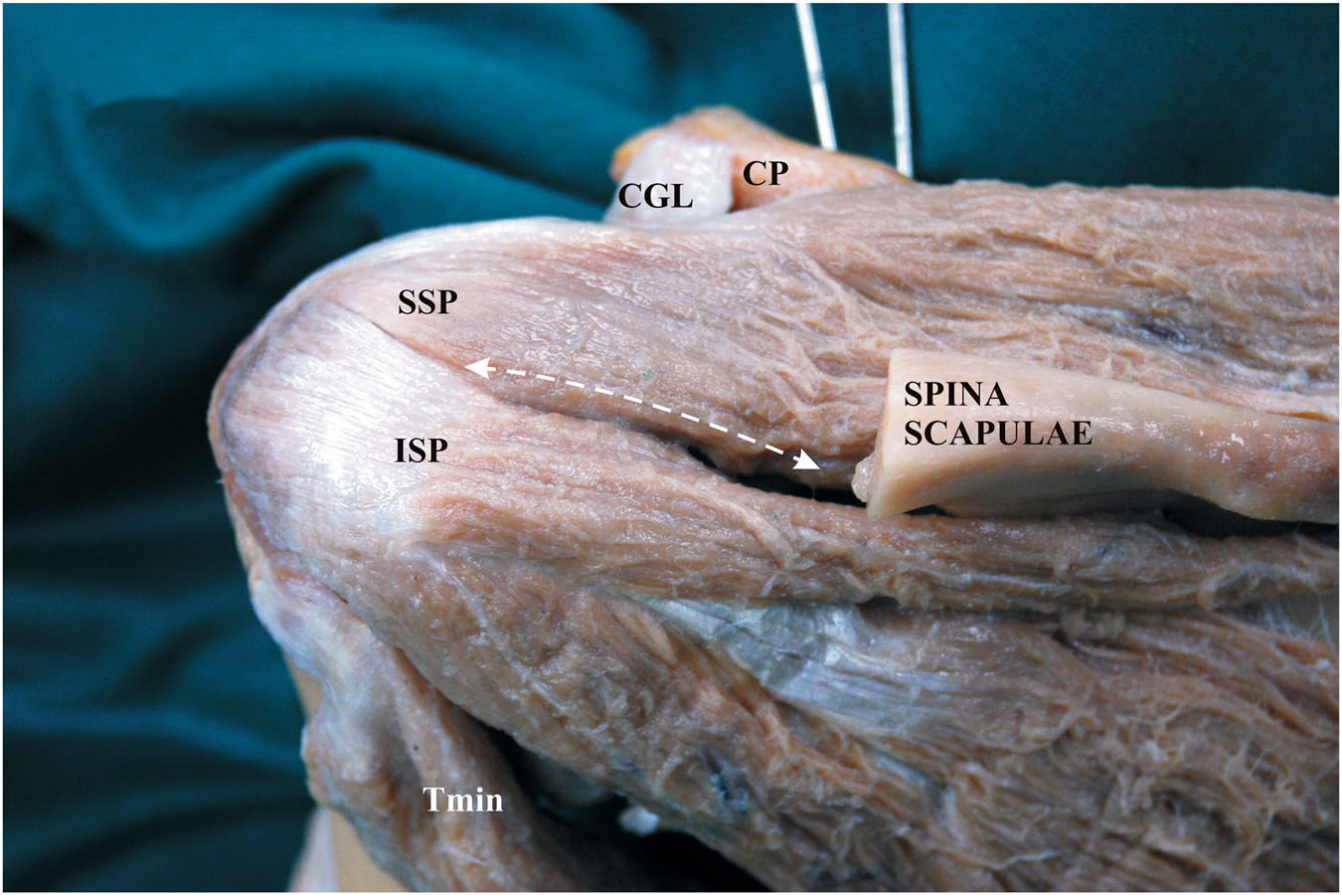
Posterosuperior view of a dissected specimen of the left shoulder joint, showing the posterior rotator interval. The acromion has been divided at the level of the spina scapulae. The posterior rotator interval (white arrow) is located between the supraspinatus (SSP) and infraspinatus (ISP) muscles. Other structures include the coracoid process (CP), coracoglenoidal ligament (CGL) and teres minor (Tmin) muscle.

**Figure 2:**
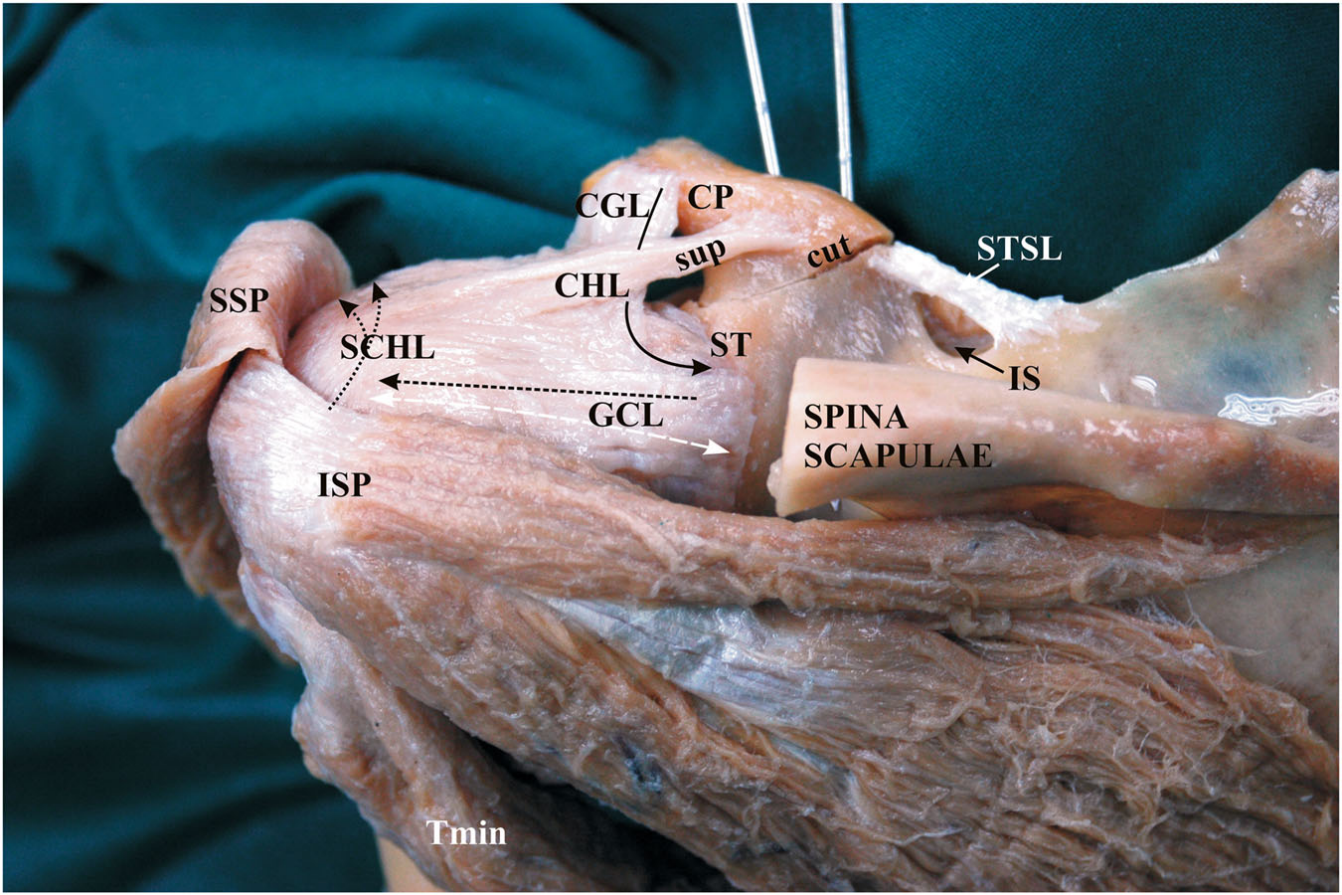
Posterosuperior view of the same dissected specimen as in Figure 1 of the left shoulder joint with attached capsular structures. The supraspinatus (SSP) muscle has been separated from the shoulder joint capsule and is located laterally. The acromion has been excised, and the coracoid process (CP) has been removed. The posterosuperior part (single black arrow) of the glenocapsular ligament (GCL) is located at the posterior rotator interval (white arrow). This ligament inserts into the humeral semicircular ligament (SCHL). Other structures include the infraspinatus (ISP), and teres minor (Tmin) muscles, supraglenoid tubercle (ST), incisura scapulae (IS), superior transverse scapular ligament (STSL), coracoglenoidal ligament (CGL) and the superior part of coracohumeral ligament (CHL) (sup).

**Figure 3:**
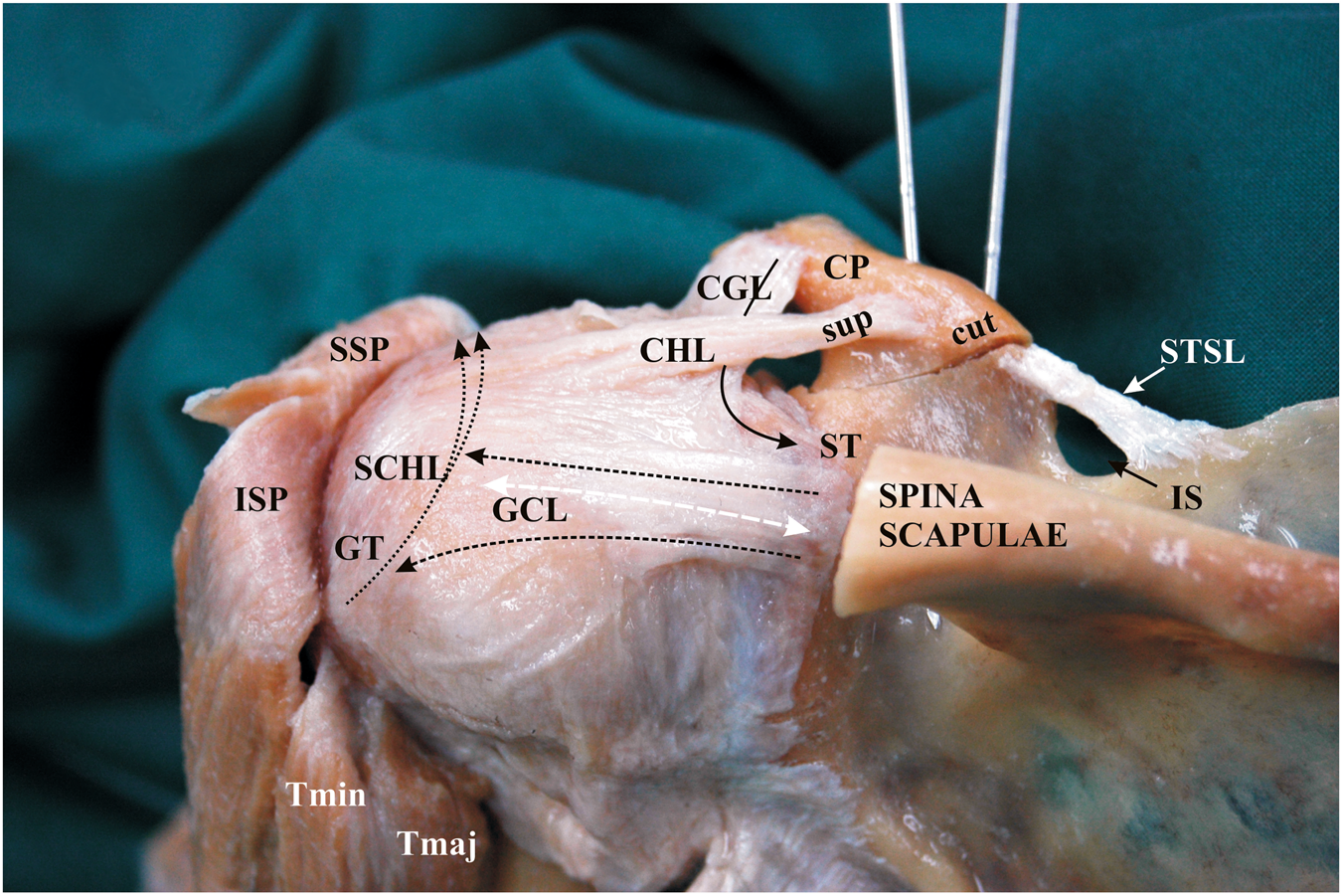
Posterosuperior view of the same dissected specimen as in Figures 1 and 2 of the left shoulder joint with attached capsular structures. The separated supraspinatus (SSP), infraspinatus (ISP), teres minor (Tmin) and teres major (Tmaj) muscles from the shoulder joint capsule are located laterally. The middle portion of the posterosuperior part (paired black arrow) of the glenocapsular ligament (GCL) is located at the posterior rotator interval (white arrow). Its attachment sites are located medially to the supraglenoid tubercle (ST)/neck of the scapula and laterally to the humeral semicircular ligament (SCHL) between the middle and inferior facets of the greater tubercle (GT) of the humerus. Other structures include the incisura scapulae (IS), superior transverse scapular ligament (STSL), coracoglenoidal ligament (CGL) and the superior part of the coracohumeral ligament (CHL) (sup).

**Figure 4:**
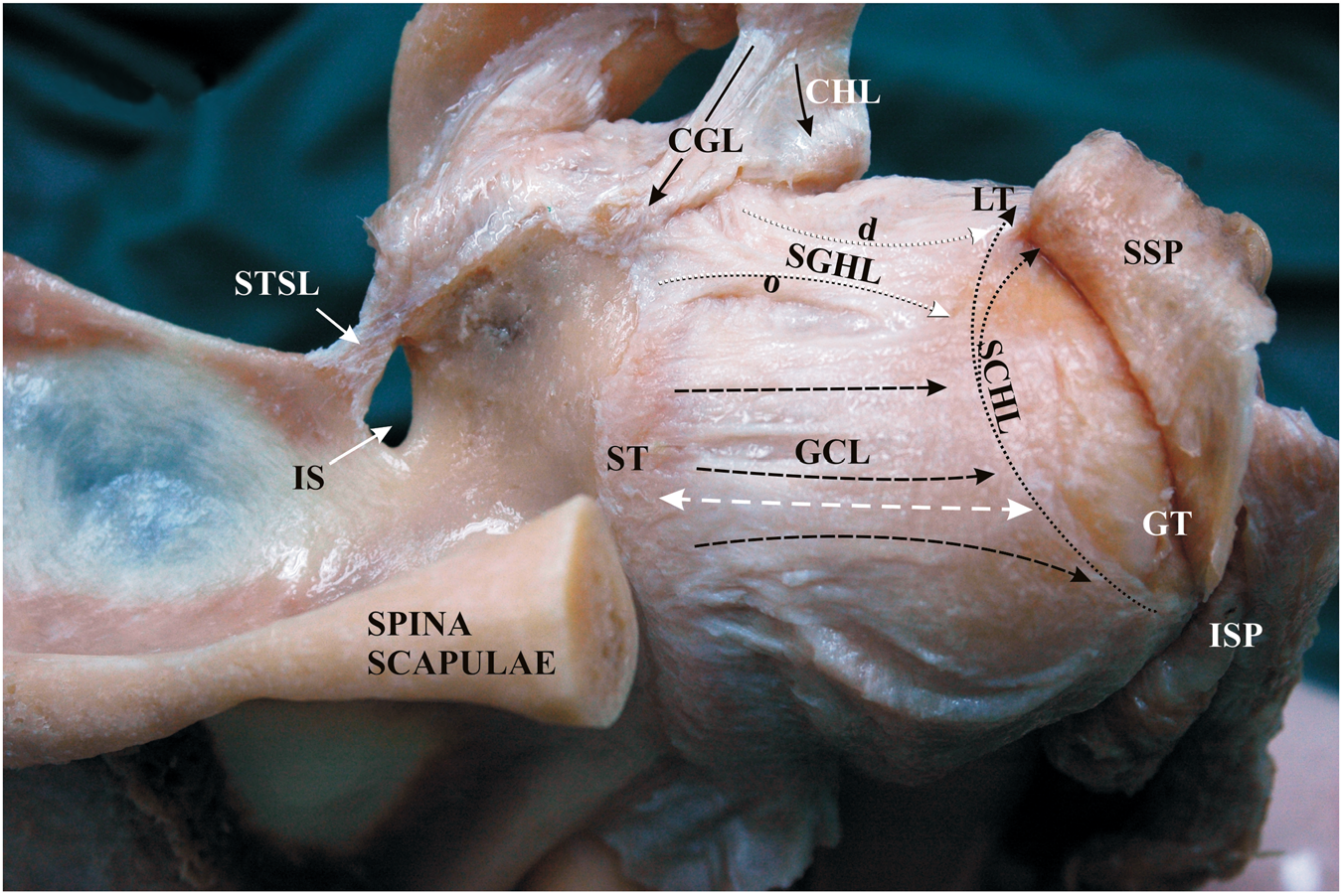
Superior view of the dissected specimen of the right shoulder with capsular structures. The rotator cuff muscles (SSP—supraspinatus; ISP—infraspinatus) have been separated from the shoulder joint capsule and are located laterally. The glenocapsular ligament (GCL) consists of the posterosuperior (black paired arrow) and mediosuperior (black single arrow) parts. The middle portion of the posterosuperior part of this ligament is located at the posterior rotator interval (white arrow). Other structures include the supraglenoid tubercle (ST), greater tubercle (GT), lesser tubercle (LT), incisura scapulae (IS), humeral semicircular ligament (SCHL), superior transverse scapular ligament (STSL), coracoglenoidal ligament (CGL) and the superior part of the coracohumeral ligament (CHL) (sup).

In all dissected shoulders, the posterior rotator interval was visible as a distinct area between the supraspinatus and infraspinatus muscles medially and laterally, where the tendons and capsular tissue fuse (Figure 1). The superficial and deep structures located at the posterior rotator interval are in line with the scapula (Figures 1, 2, 3). In all specimens, the glenocapsular ligament was found in the joint capsule at the posterior rotator interval (Figures 2, 3, 4). The glenocapsular ligament was shown to consist of either a single or two parallel‐running bundles of connective tissue fibres called the posterosuperior and mediosuperior parts (Figure 4). The posterosuperior part was constant in all specimens, whereas the mediosuperior part varied in shape and was absent in 7 of the 17 specimens (41%) (Figures 2, 3, 4). The posterosuperior part of the glenocapsular ligament was located in the posterior rotator interval, attaching medially to the supraglenoid tubercle/scapular neck and laterally to the semicircular humeral ligament (rotator cable) between the middle and inferior facets of the greater tubercle of the humerus. Moreover, the middle and posterior parts of the rotator cable were covered by the supraspinatus/infraspinatus tendons, where they fuse at the posterior rotator interval (Figures 3 and 4).

## DISCUSSION

The most important aspect of the present study is the visual description of the capsuloligamentous structures of the posterior rotator interval of the shoulder.

The glenocapsular ligament was shown to be a constant connective tissue structure consisting of either one or two different parts—the mediosuperior and posterosuperior parts. It is interesting to note that in seven shoulders, the mediosuperior part was found to be absent, while the posterosuperior part was found in all specimens, located in the posterior rotator interval in all specimens.

Miller et al. [12] have defined the existence of two different rotator intervals—the anterior and posterior intervals—while they mainly described the anatomy of the posterior rotator interval. Huri et al. [5] have explained that some distinct rotator cuff tear patterns can be associated with concomitant rotator interval injuries due to the anatomical proximity of these two anatomical regions. To avoid any misunderstanding in the current study, the terms anterior and posterior rotator interval have been used.

Miller et al. [12] have indicated that the posterior rotator interval is a consistently defined structure between the supraspinatus and infraspinatus muscles, consisting of the glenohumeral capsule medially, which fuses with the supraspinatus and infraspinatus tendons laterally. The average length of the posterior rotator interval was 77.8 mm from the spine to the tendon insertion to the humeral head in their study. The results of Miller et al. [12] did, however, not show the capsular ligamentous structures located at the posterior rotator interval, as these authors described histological fibroadipose tissue. Perhaps the ligamentous structure in this interval was unknown in their study, and therefore, biopsies for histological analysis were not taken from the ligamentous part. In contrast, the earlier findings by our research group [16] confirm that the posterosuperior part of the glenocapsular ligament is a constant connective tissue structure and is located at the posterior rotator interval in all shoulder specimens.

The findings of the current study are in line with Kolts [7], who first described the glenocapsular ligament as a connective tissue structure between the rotator cable and the cavitas glenoidale. Põldoja et al. [16] described the two anatomically different parts—the mediosuperior and posterosuperior parts of the glenocapsular ligament—and their blood supply in further detail.

Pouliart et al. [17] described a new anatomical structure at the posterosuperior part of the shoulder capsule and named this the ‘posterosuperior glenohumeral ligament’. These authors noted the importance of this ligament of the shoulder in the pathology involved in anterosuperior and posterosuperior impingement and articular‐sided rotator cuff tears as well. This also has clinical implications for rotator cuff interval and biceps pulley lesions. According to these authors, the function of this ligament, together with the coracohumeral ligament, is to anchor the rotator cable medially to the cavitas glenoidale and to help depress and centralise the humeral head and support the rotator cable function.

The present study, therefore, partly supports previous research, as these authors found a distinct longitudinal fibrous structure located at the posterosuperior part of the capsule in most specimens. The results of the present study show that this ligament consists of two parts: one part is constant and located only in the posterior rotator interval, and the other part is absent in approximately 40% of the dissected shoulders. In harmony with these findings, the different parts of the glenocapsular ligament may have distinct and different functions and varying clinical relevance.

The results of the present study are in line with earlier observations by Põldoja et al. [16], who described the glenocapsular ligament insertion area on the greater tubercle of the humerus. Both parts, the mediosuperior and the posterosuperior parts of the glenocapsular ligament, are inserted to the rotator cable in the area between the middle and inferior facets of the greater tubercle, posterior to the coracohumeral ligament.

According to Rahu et al. [18], the supraspinatus tendon was partially covered laterally by the infraspinatus tendon and fused to it in this region. The results of the present study show that the rotator cable is covered largely by the articular part of the supraspinatus tendon. The current findings support the ‘suspension bridge’ theory of Burkhart et al. [2] for rotator cuff tears and also the biomechanical function of the bridge. The glenocapsular ligament, attaching medially to the supraglenoid tubercle/scapular neck and laterally to the humeral semicircular ligament form the posterior tower of the ‘suspension bridge’. Damage to the posterior rotator cable and glenocapsular ligament damage will therefore lead to irreparable infraspinatus damage and migration of the humeral head posteriorly. If the anterior and posterior rotator cable attachment areas and the ‘suspension bridge’ tower are intact and functioning well, shoulder function may be maintained and, therefore, surgical repair with anatomical fixation may not be needed. If the rotator tendons' attachment areas are damaged anteriorly and/or posteriorly without serious anterior and posterior rotator interval damage, it is possible to restore rotator tendon integrity anatomically. However, if the ‘towers’ are destroyed, the rotator tendon damage is massive and anatomically irreparable.

### Limitations

There are some limitations to this study. The shoulder specimens analyzed were obtained from elderly donors; therefore, the findings may not be entirely generalizable to a younger population. Another limitation is the absence of quantitative measurements of length, area, and volume, as this was purely a descriptive study. No power analysis was performed, as no comparative analyses were conducted. Moreover, no histological analyses were performed to assess the quality of the ligament tissue.

## CONCLUSIONS

The posterosuperior part of the glenocapsular ligament was shown to be a constantly found connective tissue structure of the posterosuperior capsule of the shoulder joint at the posterior rotator interval. The glenocapsular ligament may be of potential clinical relevance as an anatomical reference, serving as a posterior landmark for superior capsule restoration and as an anterior landmark for supraspinatus and infraspinatus tendon restoration in large rotator cuff tears.

## AUTHOR CONTRIBUTIONS

*Cadaveric dissection, study design, writing and editing*: Elle Põldoja. *Data analysis and writing*: Tiina Tikk. *Critical reading and writing*: Jüri‐Toomas Kartus and Jon Karlsson. *Cadaveric shoulder collection*: Imke Weyers. *Cadaveric dissection and study design*: Ivo Kolts. *Study design and writing*: Madis Rahu.

## CONFLICT OF INTEREST STATEMENT

The authors declare no conflicts of interest.

## ETHICS STATEMENT

The ethical approval was required for this cadaveric study by Gesetz über das Leichen, Bestattungs‐ und Friedhofwesen des Landes Schleswig˗Holstein vom 04.02.2005, Abschnitt II, §9 (Leichenöffnung, anatomisch).

## Data Availability

The data are available from the corresponding author upon reasonable request.
